# Direct single-shot phase retrieval from the diffraction pattern of separated objects

**DOI:** 10.1038/ncomms10820

**Published:** 2016-02-22

**Authors:** Ben Leshem, Rui Xu, Yehonatan Dallal, Jianwei Miao, Boaz Nadler, Dan Oron, Nirit Dudovich, Oren Raz

**Affiliations:** 1 Department of Physics of Complex Systems, Weizmann Institute of Science, Rehovot 76100, Israel; 2 Department of Physics and Astronomy and California NanoSystems Institute, University of California, Los Angeles, California 90095, USA; 3 Department of Computer Science and Applied Mathematics , Weizmann Institute of Science, Rehovot 76100, Israel; 4 Department of Chemistry and Biochemistry, University of Maryland, College Park, Maryland 20742, USA

## Abstract

The non-crystallographic phase problem arises in numerous scientific and technological fields. An important application is coherent diffractive imaging. Recent advances in X-ray free-electron lasers allow capturing of the diffraction pattern from a single nanoparticle before it disintegrates, in so-called ‘diffraction before destruction' experiments. Presently, the phase is reconstructed by iterative algorithms, imposing a non-convex computational challenge, or by Fourier holography, requiring a well-characterized reference field. Here we present a convex scheme for single-shot phase retrieval for two (or more) sufficiently separated objects, demonstrated in two dimensions. In our approach, the objects serve as unknown references to one another, reducing the phase problem to a solvable set of linear equations. We establish our method numerically and experimentally in the optical domain and demonstrate a proof-of-principle single-shot coherent diffractive imaging using X-ray free-electron lasers pulses. Our scheme alleviates several limitations of current methods, offering a new pathway towards direct reconstruction of complex objects.

## Abstract (web summary)

Short X-ray pulses from free-electron lasers enable coherent diffractive imaging of noncrystalline objects such as single molecules. Here, the authors reconstructing full image information from a single-shot diffraction pattern by using two sufficiently separated objects to act as references for each other.

## Graphical abstract

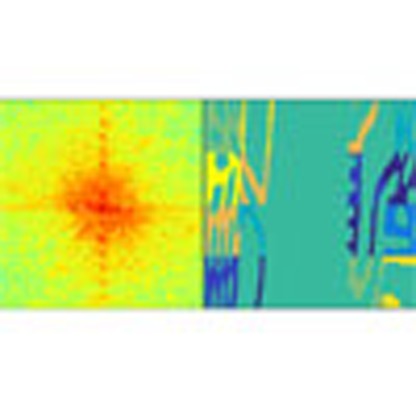

Reconstructing the phase of a field from intensity measurements is an old and ubiquitous challenge, known as the phase retrieval problem^1^,^2^,^3^. It has found numerous applications spanning from nature's smallest scales to the largest: from quantum physics^4^, material science^5^ and biology^6^, to communications and astronomy^7^. An important branch of applications is high-resolution imaging, the importance of which to physics, material science and biology cannot be overestimated. The fundamental bound on resolution—the diffraction limit—implies that imaging from a distance with subnanometric resolution requires short-wavelength sources. Since suitable lenses are not available at the very-short-wavelength (X-ray) regime, retrieval of the Fourier phase is of crucial importance. Specifically, an extremely promising application is ‘diffraction before destruction' experiments. Recent progress in X-ray sources such as X-ray free-electron lasers (XFELs) has provided ultra-bright X-ray pulses, which are as short as few femtoseconds. These pulses are bright enough to scatter a considerable amount of light from a single molecule, and fast enough to do it long before it starts to dissociate. Such ‘diffraction before destruction' methods^8^,^9^,^10^,^11^ are inherently restricted to a single diffraction pattern from every object. To reconstruct the object, the Fourier phase has to be retrieved from this diffraction pattern alone. In two and three dimensions, with sufficient oversampling, the phase retrieval problem is known to have a unique solution if the object has a finite extent (denoted as its compact support)^12^,^13^. However, retrieval of the phase is a non-convex, challenging computational task, which has been the subject of extensive study^2^,^14^,^15^,^16^,^17^,^18^,^19^. For decades, phase retrieval relied mainly on non-convex, iterative, alternating projection (AP) algorithms^1^,^2^. Although successful, AP algorithms have certain limitations, in some cases they may stagnate, in particular for complex-valued (phase) objects^20^. An alternative approach for retrieving the phase is holography, in which the unknown scattered wave is interfered with a known reference wave. In this case, the phase is mapped to an amplitude modulation, and can be uniquely retrieved by a straightforward calculation. When applicable, Fourier holography techniques^21^,^22^,^23^,^24^,^25^ offer a single-shot, direct phase reconstruction that avoids the convergence and stagnation issues of AP algorithms. Unfortunately, the generation of a well-characterized reference wave can be a difficult, and in some cases infeasible, task. Other phase retrieval schemes require several exposures of the same object, such as ptychography^26^ and the recently introduced double-blind Fourier holography (DBFH)^27^. These approaches, however, are not applicable for single-shot phase retrieval.

Here we introduce a novel lensless imaging method, which we demonstrate in 2D. This method enables phase retrieval from a single diffraction pattern via a convex approach. We show that when the single measured diffraction pattern is obtained from two (or more) sufficiently separated objects, the phase problem can be reduced to a set of linear equations that can be efficiently solved using standard numerical algebra tools. Our method does not require a careful tuning of the distance between the two objects or *a priori* knowledge of their exact support shape. It is also applicable in one dimension (1D) and can be used for phase retrieval in a wide range of applications. Moreover, it is suitable for a particularly challenging phase retrieval application, XFEL single-shot ‘diffraction before destruction' experiments. In XFEL experiments, the objects are randomly distributed, and measurements of several objects in a single-shot are common^8^,^10^. XFEL experiments are very challenging and impose quite a few technical difficulties besides phase retrieval. Notable ones are the central missing information typically due to a beam stopper, and the use of several combined detectors with missing stripes in between.

To demonstrate our method for phase retrieval, unobstructed by those additional challenges, we present a numerical study demonstrating that our method is robust to noise, as well as a reconstruction of a complicated, complex-valued object. We perform experimental reconstructions in the optical regime for both real-valued and phase objects. Finally, we show that current XFEL experiments contain the data required for our scheme (two, or more, sufficiently separated objects) by performing a proof-of-principle reconstruction of nanocrystals in the X-ray regime using XFEL pulses.

## Results

### Method description

Our scheme relies on the fact that the Fourier transform of the diffraction intensity measurement is the autocorrelation of the object. In the case of two sufficiently separated objects, their autocorrelation and cross-correlations are spatially distinct. Utilizing this, our method consists of three main steps:

(i) The sum of the objects autocorrelations, as well as their cross-correlation, are reconstructed from the Fourier transform of the measured diffraction pattern. (ii) The individual objects autocorrelations are reconstructed from their sum and the cross-correlation. (iii) Using the two intensities and the interference cross term as in refs 27, 28, 29, DBFH is applied to recover the phase by solving a set of linear equations.

**Figure 1:**
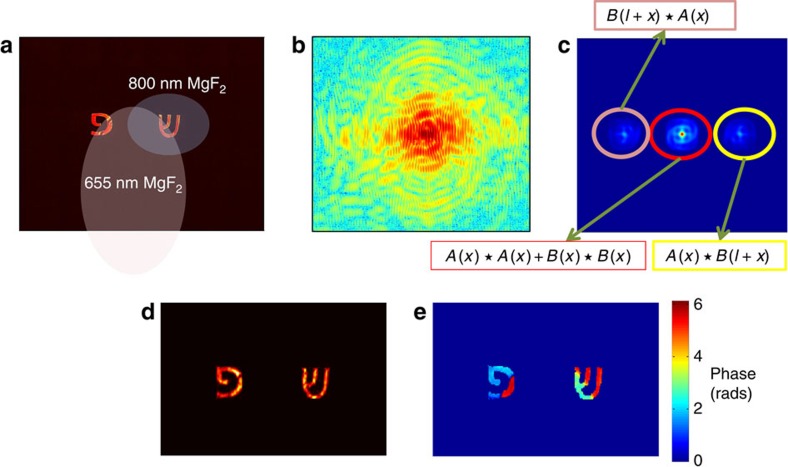
Experimental demonstration in the optical domain with two well-separated objects. (**a**) Two spatially separated objects milled into thin metal with two thin MgF_2_ films generating non-trivial phases. (**b**) The measured diffraction pattern of the two objects (in logarithmic scale). Since the objects have non-trivial phases, the diffraction is non centrosymmetric. (**c**) The two-object autocorrelation obtained by a 2D Fourier transform of the measured diffraction. Note that it can be spatially separated into the sum of single-object autocorrelations and cross-correlations. (**d**) Intensity reconstruction. (**e**) Phase reconstruction. The phase is plotted only at pixels for which the intensity is larger than ∼11% of the maximal intensity. The phase jumps inside each object, as well as the phase differences, between the two objects are in reasonable agreement with the MgF_2_ thickness.

Details of the scheme follow: consider two objects as in Fig. 1a denoted *A*(**x**) and *B*(**x**). Their Fourier spectra are given by 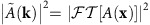 and 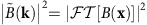, where 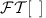 is the 2D Fourier transform operator. We define their spectral phases, 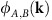 through 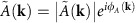 and 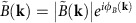. We assume that both *A*(**x**) and *B*(**x**) have a finite extent and that the two object's centres are separated by a vector **l** whose length is larger than either support width, as measured along the separation direction. The diffraction pattern of the two objects and its inverse Fourier transform, denoted 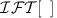, are presented in Fig. 1b,c, respectively. The latter, which is the autocorrelation of the signal, can be written explicitly as:


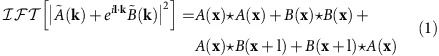


where ★ is the 2D cross-correlation operator. As can be seen in Fig. 1c the cross-correlations and autocorrelations, corresponding to the terms in equation (1), are spatially separated. Therefore, we can Fourier transform each term separately deducing the following three quantities: 
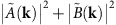
, 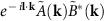 and 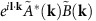, where 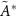 and 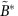 are the complex conjugates of 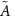 and 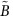, respectively.

In the second step of the algorithm, we extract the spectrum of each object, 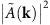 and 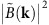 separately. First, note that


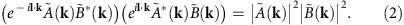


Hence, at this stage, we have recovered both the sum and the product of 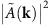 and 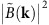, and can therefore calculate them for each value of **k** separately. However, since the sum and the product are both symmetric to exchange between 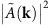 and 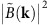, we need to identify, for each **k**, which of the two solutions is associated with 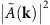 and which with 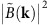. We can formulate this identification problem by introducing the following difference function:


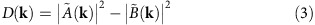


where we note that |*D*(**k**)| can be directly computed from the measured data. Clearly, by its construction, *D*(**k**) is real-valued, and identifying 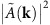 and 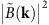 is equivalent to determining its sign. Moreover, as it is the difference between the spectra of two objects with finite extents, its inverse Fourier transform has a compact support as well. This reduces the identification problem to finding the signs of a real-valued function whose inverse Fourier transform has a compact support. A similar ‘sign problem' was studied by G. Thakur^30^, who proved that if 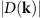 is sampled at high enough rate, then it has a unique solution even for 1D signals. We recover the sign using a novel algorithm which is applied directly in 2D, where we exploit the fact that a sign change can occur only when 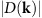 passes through a minimum. This allows us to define regions in which the sign is uniform, markedly reducing the number of unknowns. The sign is then recovered by solving an overdetermined set of linear equations (Supplementary information). After reconstructing 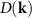, 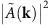 and 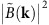 are calculated from their difference and sum, and we obtain 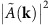, 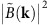 and 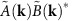. This information is sufficient for implementation of DBFH to reconstruct the objects using linear algebra^28^,^29^. For completeness, a detailed description of the DBFH scheme used in this work is presented in the Supplementary Information. In practice, the linear equations described above are set as a minimization problem of a convex functional, details of the implementation are found in the Supplementary Information. We note that, while in Fourier holography, the resolution is typically limited by the nanofabrication of the reference scattering object^22^; in our scheme, the scattering object is another (unknown) object. As a result, the resolution in our method is limited only by the aperture of the recording device and by noise. In the noiseless case, the resolution limit of our method is set solely by the aperture. In the practical, noisy case, the resolution is also limited, as in any phase retrieval method, by distortion of the reconstructed object due to noise. No nanofabrication is required and a similar nanocrystal or molecule can be used.

**Figure 2:**
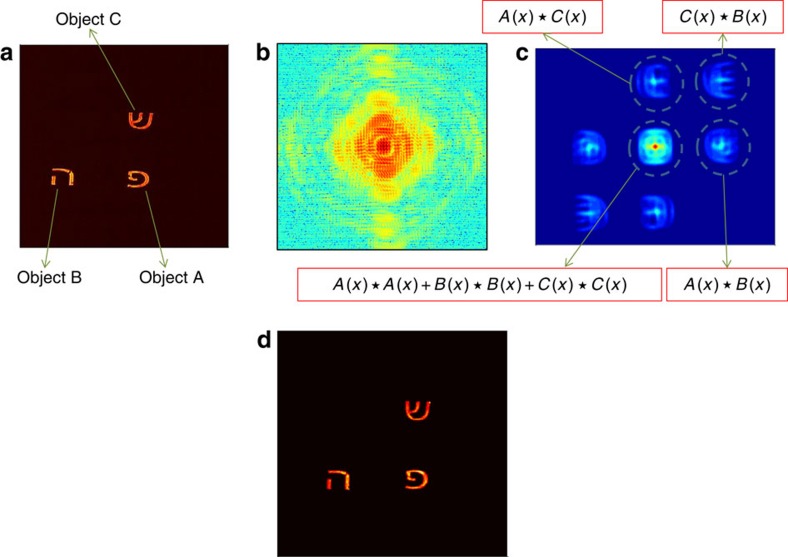
Experimental demonstration in the optical domain for three objects. (**a**) Image of the three separated objects. (**b**) The measured diffraction pattern (in logarithmic scale). (**c**) The autocorrelation (obtained by Fourier transforming the diffraction pattern) is composed of seven spatially separated components: the central one is the sum of the three single-object autocorrelations. The other six parts are the two-object cross-correlations. For clarity, three of them are explicitly described. (**d**) Intensity reconstruction from the measured scattering of the three separated objects.

Our method can also be applied to reconstruct more than two objects. We demonstrate this for the case of three well-separated objects. In this case, the algorithm can be simplified. The sign retrieval which constitutes the second part of our algorithm, can be replaced by a straightforward calculation of the single-object intensity. To see how the second step of our algorithm can be simplified, note that as described in Fig. 2, for proper distances between the three objects it is possible to resolve by spatial separation not only 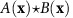 but also 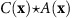 and 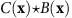, where *C*(**x**) is the third object. Their Fourier transforms are 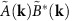, and 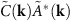 and 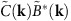, respectively. From these measurements a single-object intensity can be calculated according to:


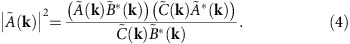


The values of 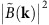 and 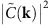 can be calculated in a similar way, without the need to solve the sign ambiguity problem.

### Experimental results

We first performed an experimental reconstruction in the optical regime. We built an experimental system using HeNe laser, measuring both the real-space image of each object and its diffraction pattern. Figure 1a shows the two objects mask, a metallic transmission plate with attached glass windows on which 800- and 655-nm films of MgF_2_ were deposited, generating non-trivial phase shifts. The acquired diffraction pattern can be seen in Fig. 1b. Note that the measured scattering is non centrosymmetric, indicating that it is indeed a complex-valued object. Also, observable is the fringe pattern typical to a pair of separated objects. Both amplitude and phase reconstructions are in very good agreement with the real-space images. Figure 2 depicts the reconstruction from three separated objects and as can be seen the agreement between the reconstruction and real-space images is very good. The intermediate results of the reconstructed individual autocorrelations of the two objects can be seen in the Supplementary Information.

After establishing our method in the optical domain where its validity can be independently verified, we show that the data needed for our method, that is, two (or more) sufficiently separated objects, are available in current XFEL experiments. To show this, we retrieve the phase of the diffraction pattern of two nanocrystals measured with a single XFEL pulse. The experimental data includes practical challenges such as central missing information^31^, systematic detection noise and the use of combined detectors with missing stripes in between them^10^. Details of the experimental system used to obtain the diffraction patterns can be found in ref. 10, and are summarized in the Methods section.

**Figure 3:**
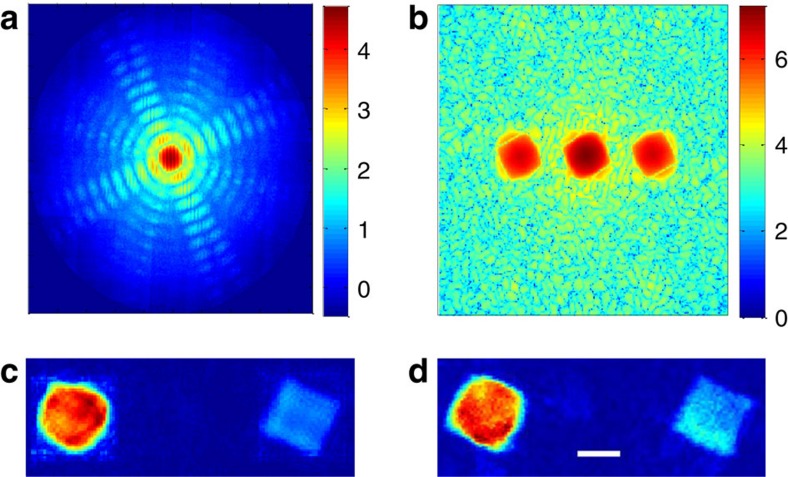
Demonstration in the X-ray domain. (**a**) An XFEL diffraction pattern of two nanocrystals (in logarithmic scale) after retrieval of the missing information and data processing (Methods). The spatial resolution of the diffraction pattern at the edge is estimated to be ∼6.5 nm. (**b**) Autocorrelation −2D Fourier transform of the diffraction pattern. (**c**) Reconstructed object with single-shot DBFH. (**d**) oversampling smoothness (OSS) reconstruction. Scale bar, 50 nm.

Figure 3a,b shows the diffraction pattern and its autocorrelation function, respectively, after reconstruction of the missing information and binning (Methods and Supplementary Information). Since the oversampling ratio of the diffraction pattern is very large^14^, we perform binning of the diffraction intensity by integrating 4 × 4 pixels into 1 pixel^32^ to reduce noise. The spatial resolution of the diffraction pattern at the edge is estimated to be 6.5 nm. We estimated the average noise level at different regions of the diffraction (Supplementary Information). The average signal at the edge of the diffraction pattern is dominated by noise; in a circle of radius ∼100 pixels, the estimated noise level is 0.3, whereas in the centre the estimated noise level is 10^−3^.

The reconstruction using our proposed method is shown in Fig. 3c. Figure 3d depicts an over sampling smoothness (OSS) reconstruction^14^ for comparison. It presents the average over the 5 best reconstructions out of 100 independent OSS reconstructions. As can be seen in Fig. 3, our reconstruction is in reasonable agreement with the OSS reconstruction. In addition to a thorough treatment of the XFEL experiments challenges described above, this implementation of our method can be significantly improved by optimizing future experimental setups. Since our method uses the interference between the nanocrystals directly, the presence of a weakly illuminated object decreases the SNR significantly. In future experiments, increasing the lateral beam size and having higher oversampling, will markedly improve the reconstruction efficiency. However, this experiment demonstrates that indeed the data required for our method is inherently present in current XFEL experiments.

We stress at this point that since our reconstruction is based on minimizing a convex functional, a single reconstruction is found, in contrast to AP methods in which multiple reconstructions using different initial conditions are computed.

### Noise stability

**Figure 4:**
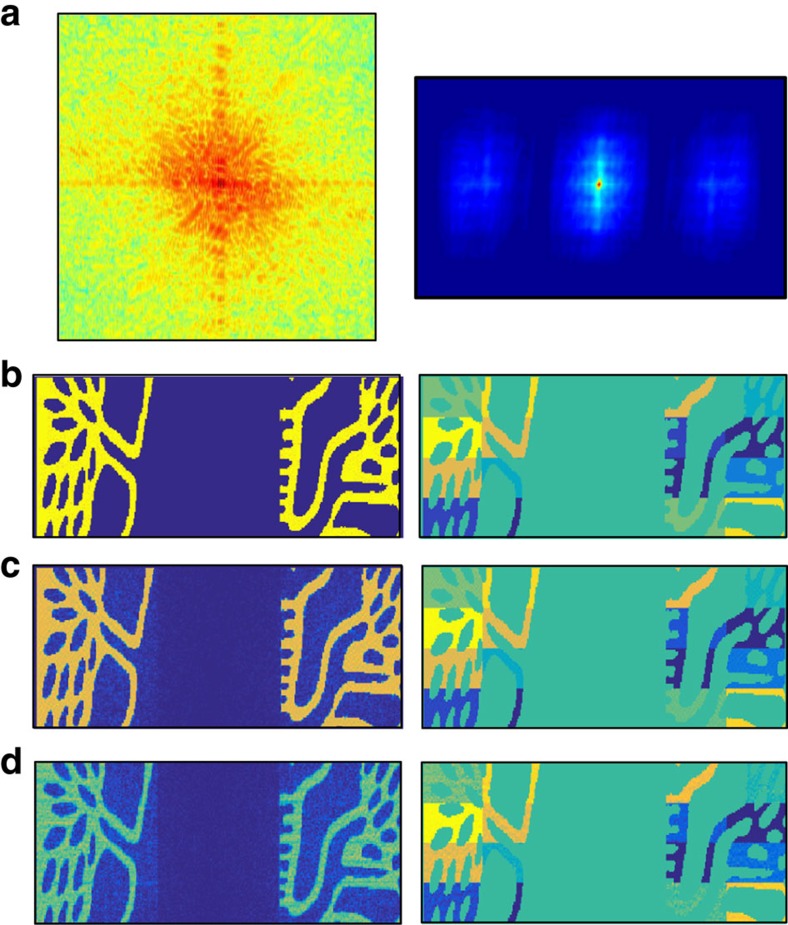
Numerical reconstruction of a complex-valued tree-shaped object from a noisy diffraction pattern. The object size is 100 × 301 pixels consisting of two 100 × 100 parts separated by 101 pixels. (**a**) Left—diffraction pattern. Right—autocorrelation. (**b**) Left—magnitude of the true object. Right—sine of the true object phase. (**c**) Noise level *σ*=10^−3^. Left—magnitude of the reconstructed object. Right—sine of the reconstructed object phase. (**d**) Same as in **c** with noise level *σ*=10^−2^.

**Figure 5:**
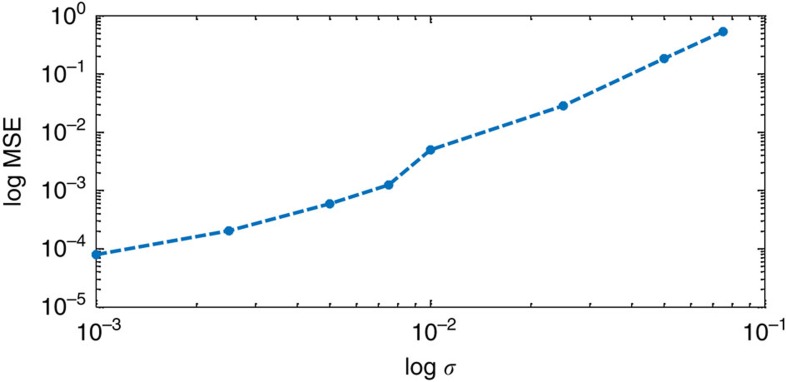
Mean squared error in different noise levels. Each point is the median of 15 noise realizations. The object consists of two 10 × 10 pixels squares with 11 pixels separation. The value of each object pixel is randomly drawn and is complex-valued.

We performed numerical simulations to further demonstrate the ability of our method to reconstruct complex objects under noisy conditions. To visualize the effect of noise on the reconstruction, we first reconstructed a complicated, complex-valued object from its noisy diffraction pattern under different noise levels (Fig. 4). In addition, we performed investigation of the noise robustness of our method using Monte Carlo simulations (Fig. 5). The object reconstructed in Fig. 4 consisted of a tree-shaped amplitude mask with a phase pattern imposed on it. The tree-shaped mask overall size was 100 × 301 pixels and it consisted of two 100 × 100 size parts separated by 101 pixels. The noise levels for the reconstruction were *σ*=10^−3^ and 10^−2^ (the noise model was as in ref. 29). As can be seen in Fig. 4, the reconstruction is in good agreement with the true object both in magnitude and phase. The effect of increasing the noise level is apparent, variations arise in the magnitude of the noisy reconstructed objects although the objects shape is well preserved, milder phase variations are also apparent. The sine of the real-space phase reconstruction is presented for amplitude >0.1 for clarity. For the Monte Carlo simulations, we used a complex-valued, randomly drawn object. It consisted of two squares of size 10 × 10 pixels with 11-pixel separation. We performed reconstructions from the noisy diffraction pattern of the object at different noise levels. For each noise level, we took the median of 15 noise realizations. We calculated the mean squared error between the absolute value of the reconstructed object and the true object. The results are presented in Fig. 5. As can be seen, the reconstruction is quite robust up to about *σ*=10^−2^ demonstrating that our method can reconstruct under noisy conditions complicated and complex-valued objects. Furthermore, the noise stability can be improved by proper weighting and noise analysis. This will be the subject of future research.

## Discussion

We have presented and experimentally demonstrated a novel direct method for single-shot phase retrieval from the diffraction pattern of at least two well-separated finite objects. By virtue of decomposing the autocorrelation function to three independent parts our method evades the non-convexity of the phase problem on the one hand, and the need for a well-characterized reference field on the other. In the noise-free case, with sufficient oversampling, the method is guaranteed to yield a unique solution (Supplementary Information) and its robustness to noise, which is numerically and experimentally demonstrated here, can be also analysed using well-established numerical algebra tools^29^. Importantly, the experimental requirement for separated objects is compatible with current ‘diffraction before destruction' schemes, where measurements of two or more particles are common. This work is, to the best of our knowledge, the first demonstration of a convex phase retrieval scheme from a single diffraction pattern, moreover, is also applicable in 1D. As such, it paves the path to numerous phase retrieval applications from coherent diffractive imaging and electron diffraction to ultrashort pulse reconstruction and quantum state tomography.

## Methods

### Optical experiment

We used a HeNe laser (*λ*=63.2 nm), and collected the scattered light from the sample through two paths. In the first path, the light was focused onto a charge-coupled device (CCD) camera, thus measuring the diffraction pattern. In the second path, the light was imaged onto another CCD camera for comparison with the reconstruction.

### XFEL experiment

The XFEL experiment was performed as detailed in ref. 10. In summary, nanocrystals were randomly positioned on a 100-nm-thick Si_3_N_4_ membrane. The single-shot exposures were conducted by focusing XFEL pulses onto a 1.5-μm spot on the membrane, and scanning the spot position. A large data set of diffraction patterns was measured from which the diffraction pattern of a pair of sufficiently separated nanocrystals was chosen for our reconstruction.

### XFEL data preparation for reconstruction

The raw data measured by a multiport CCD (octal MPCCD) and had several missing bars of width 1–6 pixels. After background subtraction, the missing bars were completed by interpolation. In the next step, 4 × 4 pixels binning was performed to reduce noise, if more than half the pixels in the binning were zero then the binned pixel was set to zero. In addition to the missing bars, the raw data contained a central missing information region of size 61 × 64 pixels. This was reconstructed using the fact that the Fourier transform of the diffraction pattern, the autocorrelation, has a finite extent. Using this information, the problem was recast as a set of linear equations solved for the missing information pixels (similarly to ref. 33). Finally, since the objects are known to be real-valued, we imposed centrosymmetry on the diffraction amplitudes.

## Additional information

**How to cite this article:** Leshem, B. *et al.* Direct single-shot phase retrieval from the diffraction pattern of separated objects. *Nat. Commun.* 7:10820 doi: 10.1038/ncomms10820 (2016).

## Supplementary Material

Supplementary Information Supplementary Figures 1-2, Supplementary Notes 1-5 and Supplementary References.
