# Supplementary material for: Direct single-shot phase retrieval from the diffraction pattern of separated objects
Source: Nat Commun. 2016 Feb 22;7:10820. doi: 10.1038/ncomms10820 (PMC4764927; doi:10.1038/ncomms10820)
Supplement: Supplementary Information — Supplementary Figures 1-2, Supplementary Notes 1-5 and Supplementary References. [file ncomms10820-s1.pdf]

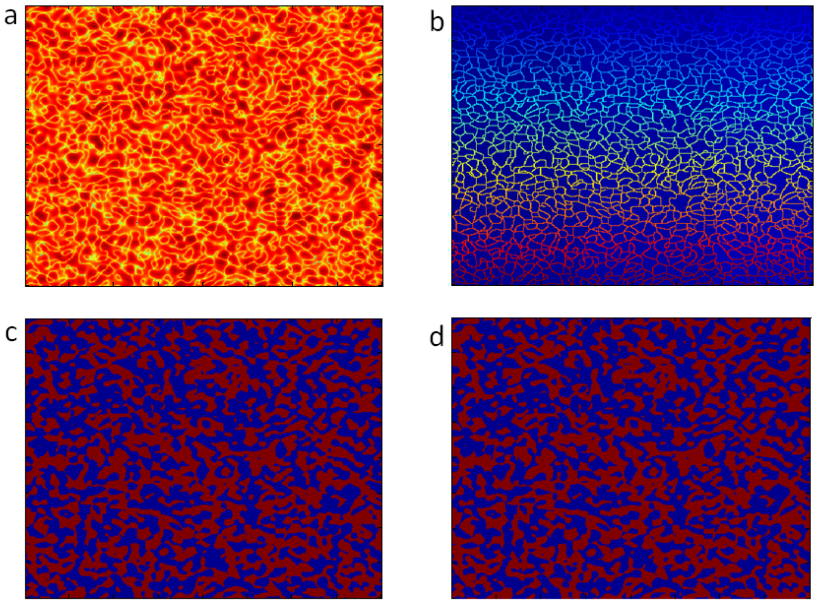


Supplementary Figure 1: **Results for the sign reconstruction from a random complex-valued object.** The object consists of a pair of $40X40$ pixels squares. a) $|D(\mathbf{k})|$ (in logarithmic scale). b) Segmentation to constant sign regions using the watershed algorithm. Each region of constant sign (as well as the pixels on the boundaries) is assigned with an integer number in ascending order (this is the color code). c) Sign reconstruction. Red denotes positive sign and blue negative. d) True sign.

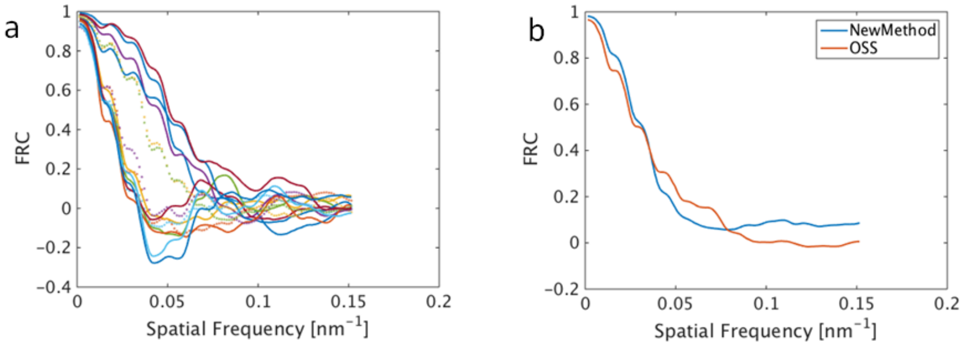


Supplementary Figure 2: **Fourier Ring Correlation (FRC).** a)Solid line - FRC between two out of the best 5 OSS reconstructions. Dotted line - FRC between the best 5 OSS reconstruction and the proposed method. b) FRC calculated between the averaged reconstruction with OSS and with our method. The spatial frequency of the diffraction pattern at the edge is 0.154 $nm^{-1}$.

## Supplementary Note 1 - Double blind Fourier holography

In a typical holography scheme, the diffracted field from an unknown object is interfered with a known reference field. Various methods exists to retrieve the unknown field from this information. In DBFH, the interference between two unknown fields is used without any known reference field. Consider two finite objects or two non overlapping parts of the same object, denoted $A(\mathbf{x})$ and $B(\mathbf{x})$. Their Fourier spectra are given by $|\tilde{A}(\mathbf{k})|^2 = |\mathcal{FT}[A(\mathbf{x})]|^2$, $|\tilde{B}(\mathbf{k})|^2 = |\mathcal{FT}[B(\mathbf{x})]|^2$ , and their spectral phases, $\phi_{A,B}(\mathbf{k})$ are given through $\tilde{A}(\mathbf{k}) = \left|\tilde{A}(\mathbf{k})\right| e^{i\phi_A(\mathbf{k})}$ and $\tilde{B}(\mathbf{k}) = \left|\tilde{B}(\mathbf{k})\right| e^{i\phi_B(\mathbf{k})}$. The input for the DBFH algorithm are the following diffraction patterns: $|\tilde{A}(\mathbf{k})|^{\mathbf{2}}$ and $|\tilde{B}(\mathbf{k})|^{\mathbf{2}}$, along with $|\tilde{A}(\mathbf{k})+\tilde{B}(\mathbf{k})|^{\mathbf{2}}$ from which the interference term $\tilde{A}(\mathbf{k})\tilde{B}^*(\mathbf{k})$ is obtained [1]. Let us denote the unknown phases as $X_A(\mathbf{k}) = e^{i\phi_A(\mathbf{k})}$ and $X_B(\mathbf{k}) = e^{i\phi_B(\mathbf{k})}$ respectively. The compact support information can be expressed by the following equations (for $\mathbf{x}$ *outside* the compact support):

$$\mathcal{IFT}\left[|\tilde{A}(\mathbf{k})|X_A(\mathbf{k})\right](\mathbf{x}) = 0 \ \ (\forall \mathbf{x} \notin C.S) \tag{1}$$

$$\mathcal{IFT}\left[|\tilde{B}(\mathbf{k})|X_B(\mathbf{k})\right](\mathbf{x}) = 0 \ \ (\forall \mathbf{x} \notin C.S) \tag{2}$$

Supplementary Eqs. 1 and 2 yield an under-determined set of equations since there are more variables (the overall number of pixels) than equations (only the pixels outside the compact support). However, using the interference term, additional equations are obtained:

$$\tilde{A}(\mathbf{k})\tilde{B}(\mathbf{k})^* X_B(\mathbf{k}) = \left|\tilde{A}(\mathbf{k})\right|\left|\tilde{B}(\mathbf{k})\right| X_A(\mathbf{k}) \tag{3}$$

Importantly, we relax the constraints that $X_A(\mathbf{k})$ and $X_B(\mathbf{k})$ are phases (namely that they have unit modulus) [2]. With sufficient oversampling (which is sampling of the Fourier plane at twice the Nyquist rate for the combined size of both objects, see [2]), Supplementary Eqs.(1-3) are overdetermined and therefore have a unique nontrivial solution (in the least squares sense). The robustness to noise is investigated in [2] and experimentally demonstrated in the optical regime in [1]. As described in [1, 2, 3], the compact support can be estimated from the measured data itself. In practice, however, a rough estimation of the supports length and width from the autocorrelation is sufficient for reasonable reconstruction and the exact support shapes is not required. The numerical solution of Supplementary Eqs.(1-3) can be performed for each row and column separately as was done in [1]. Alternatively, the equations can be solved directly in 2D, as was done here. In this case, the equations can be casted as a minimization problem:

$$\begin{aligned} &\text{minimize} \quad && \text{norm}(\mathcal{A}X - b) \\ &\text{subject to} \quad && \mathcal{C} \end{aligned} \tag{4}$$

Here, $\mathcal{A}$ is as described in [1] and $\mathcal{C}$ is some constraint set, which is null in this work. In 2D this will constitute a very large set of equations: $\mathcal{A}$ is a matrix of size $N^2X(N^2-\tau^2)$, with $N^2$ the number of pixels in the diffraction

pattern and $\tau^2$ the number of pixels in the compact support of the object. To apply this efficiently in 2D, we used Matlab's Preconditioned conjugate gradients method (PCG). Instead of solving directly a set of equations, which can be extremely computationally demanding in 2D, we use the fact the fast Fourier transform is implemented efficiently in Matlab (via fft2) to construct a function for minimization in PCG.

# Supplementary Note 2 - Solving the sign problem in 2D

In this section, we describe the sign reconstruction algorithm used in the article. The problem is to retrieve the phase of the function $D(\mathbf{k})$ with the constraints that $D(\mathbf{k})$ is real and its inverse Fourier transform has a compact support. This is a special case of the phase retrieval problem in which the phase can only take the values 1 or $-1$. Since the Fourier transform of $\mathcal{IFT}[D(\mathbf{k})]$ has a compact support, equations similar to Supplementary Eqs. 1 and 2 can be written. Denoting the unknown sign as $S(\mathbf{k})$ we have:

$$\mathcal{IFT}\left[|D(\mathbf{k})|\, S(\mathbf{k})\right](\mathbf{x}) = 0 \;\; (\forall \mathbf{x} \notin C.S) \tag{5}$$

As described in the previous section this constitutes an under-determined set of equations. However, the fact that $D(\mathbf{k})$ is real implies that the value of $S(\mathbf{k})$ in neighboring points can only change if there is a zero crossing of $D(\mathbf{k})$ between them. Furthermore, the fact the Fourier transform of $D(\mathbf{k})$ has a compact support iposes a bound to the rate in which $D(\mathbf{k})$ can change. Hence, in a region in which all the values of $|D(\mathbf{k})|$ are large enough $S(\mathbf{k})$ must be constant. This constraint yields a large number of additional linear equations:

$$S(\mathbf{k_m}) = S(\mathbf{k_l}) \;\; \mathbf{k_m}, \mathbf{k_l} \in \text{same segment}. \tag{6}$$

Combining the segmentation to constant sign regions with Supplementary Eq.5 can be shown to yield a unique solution even in 1D (with sufficient oversampling). Moreover, uncertainties in the segmentation process can be compensated for with additional oversampling. The required oversampling can be shown to be sampling $D(\mathbf{k})^{\mathbf{2}}$ at the Nyquist rate plus the number of segments. Hence, if $D(\mathbf{k})$ is of size $\tau$ and is devided to $M$ segments, the required oversampling is $2\tau + M$. The Proof of uniqueness, optimized segmentation scheme and detailed noise robustness analysis are the subject of a paper under preparation. Importantly, we stress that in Supplementary Eqs.(5) and (6) we relax the constraint that $S(\mathbf{k})$ is a sign. In practice, we used the fact that zero crossing of $D(\mathbf{k})$ occurs only in the vicinity of minima of $|D(\mathbf{k})|$ to perform the segmentation by applying the watershed transform [4]. Supplementary Fig.(1a) depicts $|D(\mathbf{k})|$ of an object consisting of a pair of square of size $40X40$ pixels with 41 pixels separation, the object is randomly drawn and is complex valued. Supplementary Fig.(1b) is its segmentation to constant sign regions. The different colors in Supplementary Fig.(1b) denote constant sign regions by ascending order. Note that the signs themselves are not presented in this figure, but only region in which the sign is known not to change. Also note that we are actually performing over-segmentation, in the sense that sometimes regions of constant sign are divided into several segments (as can be seen by comparison to Supplementary

Fig(1c) and (d)). This means that better segmentation will decrease the number of variables and improve noise stability. The reconstructed signs are presented in Supplementary Fig.(1c) and the true signs in Supplementary Fig.(1d). We note that as in the case of DBFH described above, expressing Supplementary Eqs.(5) and (6) directly in 2D is very computationally demanding. Instead we use again the PCG function, similarly to what is done for DBFH. Also, instead of adding equations as in Supplementary Eq.(6), we use the segmentation to reduce the number variable by imposing that each segment is a single variable. The number of variables was reduced in this case from 160000 to 35846 (The number of equations in this case is 158400, so the system of eqautions is vastly overdetermined). Typically, even though our segmentation process is far from optimal, it reduces the number of variables by about one order of magnitude or more.

# Supplementary Note 3 - Completing the missing pixels

In most X-ray lensless experiments, the center of the diffraction pattern cannot be measured due to the photons which do not scatter from the object. Nevertheless, as was shown in [7], with high oversampling the object can be reconstructed even when the center of the diffraction pattern is blocked. Most phase retrieval algorithm, which are based on iterative projections, handle the missing information simply by not imposing any constraint on the intensities on the missing pixels [8]. The method proposed here, however, is incompatible with this approach. A different approach to handle the missing information problem, based on linear algebra arguments, was presented in [9]. In our reconstruction we adopted a similar approach. Detailed description on our approach, including noise analysis, will be published elsewhere.

Let us denote the total number of pixels by $n$ and the number of missing pixels by $m$. The measured intensity with zeros in the missing pixels is denoted by $I(\mathbf{k})$. We denote by $M(\mathbf{k})$ the image which has the correct intensity at the missing pixels and is zero at all the measured pixels, such that $I(\mathbf{k}) + M(\mathbf{k})$ is the true intensity pattern (up to noise). The inverse Fourier transform of $I(\mathbf{k}) + M(\mathbf{k})$ is the autocorrelation of the combined object, therefore it has a compact support (which can be expressed in terms of the compact supports of the two objects and the distance between them). Outside the compact support the autocorrelation must be zero. Therefore we can write:

$$\mathcal{IFT}\left[\tilde{I}(\mathbf{k}) + \tilde{M}(\mathbf{k})\right](\mathbf{x}) = 0 \;\; (\forall \mathbf{x} \notin C.S) \tag{7}$$

The number of equations here is the number of pixels we know are outside the compact support. If this number is larger then $m$, then there are more equations than unknowns, and the missing pixels can be calculated by simple inversion. In our case the number of missing pixels is about 1257 (after binning), and the number of pixels which we know a priori to be outside of the compact support is about 230,000 , therefore our system of equations is vastly overdetermined.

## Supplementary Note 4 - Fourier Ring Correlations for the XFEL Reconstruction

To compare the X-ray reconstructions of the method proposed and the OSS reconstruction we use the Fourier Ring Correlation (FRC) method [10] which allows comparison of two images. This is done by calculating

$$FRC(k_i) = \frac{\sum_{k\in k_i} F_1(k)F_2^*(k)}{\sqrt{\sum_{k\in k_i} F_1(k)^2 \cdot \sum_{k\in k_i} F_2(k)^2}} \quad (8)$$

where $F_1(k)$ and $F_2(k)$ are the Fourier transforms of the compared images.

We used Supplementary Eq.(8) to calculate the FRC between our (averaged) reconstruction and each of the OSS reconstructions (Supplementary Fig.(2a)), as well as the FRC between the final reconstructions presented in the paper (Supplementary Fig.(2b)). In Supplementary Fig.(2a), since we compare pairs of images taken from 6 different images, there are overall 15 different FRC curves. The FRC between each pair of OSS reconstructions are plotted as solid lines, and the FRC between OSS reconstructions and our method are plotted as dots. As can be clearly seen from this image, the reconstruction with the proposed method is well within the ballpark of the OSS reconstruction. The estimated resolution based on the FRC=0.5 criterion is $\sim$ 14nm for our method and $\sim$ 18nm for OSS.

## Supplementary Note 5 - Estimation of the noise level in the XFEL experiment

An estimation of the noise level in the experimental XFEL diffraction pattern was performed for 3 regions in the diffraction pattern. The noise level was estimated as the average noise energy divided by the average signal energy as described below. First the noise was estimated by averaging over the energy in the autocorrelation outside of the compact support. Next, the signal energy over 3 different regions in the diffraction pattern was estimated by averaging the energy there. The regions used for estimation were: circle around the center, a ring around 100 pixels off the center, and a ring near the edge. Finally, the noise level at each region was estimated by dividing the estimated noise with the estimated signal at each region,

## Supplementary References


[1] Raz, O. *et al.* Direct phase retrieval in double blind fourier holography. *Opt. Express* **22**, 24935–24950 (2014).

[2] Raz, O., Nadler, B. & Dudovich, N. Vectorial phase retrieval for 1-d signals. *IEEE Trans. Sig. Proc.* **61**, 1632 – 1643 (2013).

[3] Raz, O. *et al.* Vectorial phase retrieval for linear characterization of attosecond pulses. *Phys. Rev. Lett.* **107**, 133902 (2011).

[4] Roerdink, J. B. & Meijster, A. The watershed transform: Definitions, algorithms and parallelization strategies. *Fundamenta Informaticae* **41**, 187–228 (2000).

[5] Seibert, M. M. *et al.* Single mimivirus particles intercepted and imaged with an x-ray laser. *Nature* **470**, 78–81 (2011).

[6] Faulkner, H. M. L. & Rodenburg, J. M. Movable aperture lensless transmission microscopy: A novel phase retrieval algorithm. *Phys. Rev. Lett.* **93**, 023903 (2004).

[7] Miao, J., Sayre, D. & Chapman, H. N. Phase retrieval from the magnitude of the Fourier transforms of nonperiodic objects. *J. Opt. Soc. Am. A* **15**, 1662–1669 (1998).

[8] Miao, J. *et al.* Quantitative Image Reconstruction of GaN Quantum Dots from Oversampled Diffraction Intensities Alone. *Phys. Rev. Lett.* **95**, 085503 (2005).

[9] Thibault, T. *et al.* Reconstruction of a yeast cell from X-ray diffraction data. *Acta Cryst. A.* **62**, 248–261 (2006).

[10] Saxton, WO and Baumeister, W. The correlation averaging of a regularly arranged bacterial cell envelope protein. *Journal of Microscopy* **127**, 127–138 (1982).
